# Illustrations of the Influence of the Mind upon the Body in Health and Disease

**Published:** 1873-04

**Authors:** 


*Illustrations of the Influence of the Mind upon the Body in Health and Disease. Designed to elucidate the action of the Imagination.* By DANIEL HACK TUKE, M.D., M.R.C.P. Philadelphia: Henry C. Lea, 1873. Buffalo: T. Butler & Son.

The author of this work has been for some time known as a writer and investigator in the department of pyschological medicine. The subject of the influence of the mental upon the physical organization, has ever since the infancy of medical science attracted the attention of those accustomed to the care of the sick. Various explanations have been advanced to account for many of the phenomena observed in the sick room, and many and marvelous have been the cures ascribed to witchcraft, the influence of sacred relics, and even to medicine itself, which were due wholly to the infiuence of the imagination.

The work under consideration comprises over four hundred pages and is divided into four sections, part first treating of the Intellect, to which about one hundred pages are devoted, part second the Emotions, occupying two hundred pages, part third the Will, embracing twenty-one pages, and part fourth the Influence of the Mind upon the Body in the cure of Diseases, which together with a short appendix comprising the balance of the book.

The author informs us in the introductory chapter that the "Mind acts upon the Body through its threefold states of—I Intellect, II Emotion, III Volition." The writer says "It must be clearly understood that under "Mind" we do not, and that under "Body" we do, include the special senses." He dwells upon the importance of the truth "that the mind or brain influences—excites, perverts, or depresses—the sensory, motor vasomotor, and trophic nerves, and through them causes changes in sensation, muscular contraction, nutrition and secretion."

To the want of appreciation of this truth may be ascribed the superstitious vagaries of the past when witchcraft held its powerful sway and to the touch of royalty, the influence of sacred relics, and the sprinkling of holy water , were ascribed cures wrought by faith alone.

The conclusions arrived at by the author are clearly stated and will meet the approbation of the reader who carefully follows the course of reasoning pursued.

The writer does not rely wholly upon his own observations, but from a wide range of reading and inquiry brings illustrative cases to support his arguments. The writings of other investigators of Psycho-physical phenomena are fully quoted where they will serve to aid in establishing some fact or combating some erroneous opinion.

The Influence of the Mind upon the Body is without doubt one of the strongest therapeutical agencies which can be wielded by the physician, and he who passes it by as unworthy of his attention or who uses it carelessly and in ignorance of its true scope and value, is neglecting or misusing a valuable assistant in his battle with disease and death. As the imagination may be and is a valuable assistant in the treatment of disease so also it may be a stumbling block, and what physician is there who would not rather treat a score of patients suffering from some real malady than one who continually imagines that he has all the ills to which flesh is heir, pent up in his single body. The powerful influence of the mental organization upon diseases is something which has long been noticed and taken advantage of by the quacks And although the true secret has in many instances been misunderstood, yet their incantations, relics and other absurdities have often gained the credit for miracles wrought alone by all powerful faith.

The object of the author has been not only to show the power of the mind in the cure of disease, but also to attract the attention of the profession more closely to the subject. Quackery in its many forms has for ages used and abused this agency, and it is the desire of the author to invite the attention of the profession to a subject at once so powerful for good, and so little understood, that the medical man may in the future apply in a methodical manner the principles of Psycho-therapeutics. "The force is there, acting irregularly and capraciously. The question is whether it cannot be applied and guided with skill and wisdom by the physician."

In writing this book the author has placed the profession under many obligations to him, and as the subject is more fully investigated as time passes along the reader of the future will feel with increased force his debt of gratitude to Dr. Tuke for placing in his hands a work filled with fresh and entertaining truths, stated in terms easily understood. The American publishers should receive the thanks of the profession for placing the work in their hands. We advise our readers to obtain it and study it with care.

